# Uni-port video-assisted thoracoscopic surgery for pulmonary arteriovenous malformation based on vascular pre-occlusion: a case report and literature review

**DOI:** 10.3389/fmed.2026.1918471

**Published:** 2026-08-21

**Authors:** Yanhui Yang, Jinyuan Mei, Ji Li, Xin Cheng, Min Jing, Hong Shi, Yi Wang

**Affiliations:** 1 Department of Cardiothoracic Surgery, The First People’s Hospital of Neijiang, Neijiang, China; 2 Department of Critical Care Medicine, Huashan Hospital Affiliated to Fudan University, Shanghai, China; 3 Department of Pathology, The First People’s Hospital of Neijiang, Neijiang, China; 4 Department of Ultrasound, The First People’s Hospital of Neijiang, Neijiang, China

**Keywords:** hereditary hemorrhagic telangiectasia, pulmonary wedge resection, pulmonary arteriovenous malformation, uni-portal video-assisted thoracoscopic surgery, vascular pre-occlusion

## Abstract

**Introduction:**

Pulmonary arteriovenous malformations (PAVMs) are uncommon vascular anomalies characterized by abnormal communications between pulmonary arteries and veins. Although transcatheter embolization is considered the first-line treatment for most PAVMs, surgical intervention remains an important option for selected patients, particularly those with large lesions or unfavorable anatomy. However, reports describing uniportal video-assisted thoracoscopic surgery (UVATS) for PAVM treatment remain limited. We report a case of a large PAVM successfully managed using UVATS with temporary pre-blocking of the feeding pulmonary artery and draining pulmonary vein, and review the literature regarding thoracoscopic management of PAVMs.

**Case description:**

A 43-year-old woman was admitted after a routine health examination revealed a right lower lobe pulmonary lesion. The patient was asymptomatic, and imaging studies, including contrast-enhanced computed tomography and three-dimensional reconstruction, demonstrated a 3.1 cm × 2.7 cm pulmonary arteriovenous malformation located in the outer basal segment of the right lower lobe (RS9). UVATS was performed through a single 3-cm incision. After complete hilar dissection, the right lower pulmonary artery and right lower pulmonary vein were temporarily occluded before wedge resection of the lesion. The operation was completed in 50 min without intraoperative complications. Postoperatively, room-air oxygen saturation improved from 92% to 98%, and arterial oxygen tension increased from 66 to 82 mmHg. Histopathological examination confirmed the diagnosis of PAVM. The chest tube was removed on postoperative day 2, and the patient was discharged on postoperative day 3. No recurrence or adverse events were observed during the 3-month follow-up period.

**Conclusion:**

This case demonstrates that UVATS combined with temporary pre-blocking of both the feeding and draining pulmonary vessels is a feasible and safe approach for selected patients with large PAVMs. Temporary vascular control may reduce the risks of intraoperative bleeding and embolic complications and facilitate successful minimally invasive resection. Further studies are warranted to evaluate the reproducibility and long-term outcomes of this strategy.

## Highlights

- We report the successful treatment of a large pulmonary arteriovenous malformation (PAVM) using uniportal video-assisted thoracoscopic surgery (UVATS) with temporary pre-blocking of both the feeding pulmonary artery and draining pulmonary vein before lesion resection.
- The patient recovered uneventfully, showed improved oxygenation postoperatively, and remained free of recurrence during follow-up.

What is known and what is new?

- What is known?

PAVMs are abnormal communications between pulmonary arteries and veins that may result in hypoxemia, paradoxical embolism, and life-threatening hemorrhage.

Transcatheter embolization is generally considered the first-line treatment; however, recurrence and procedure-related complications may occur.

Thoracoscopic surgery is an effective alternative treatment in selected patients, but reports of UVATS for PAVM are limited.

- What is new?

This report describes a novel UVATS strategy involving temporary pre-blocking of both the pulmonary artery and pulmonary vein prior to resection of a large PAVM.

We additionally summarize the published literature on thoracoscopic management of PAVMs, highlighting the feasibility and potential advantages of minimally invasive surgical approaches.

What is the implication, and what should change now?

- Temporary vascular pre-blocking may improve operative safety by reducing the risks of intraoperative bleeding and systemic embolization during manipulation of large PAVMs.
- UVATS should be considered a feasible minimally invasive treatment option for selected patients with anatomically suitable PAVMs.
- Further multicenter studies are warranted to validate this technique and establish its role alongside transcatheter embolization in the management of PAVMs.

## 1 Introduction

### 1.1 Background

Pulmonary arteriovenous malformations (PAVMs) are uncommon but clinically important vascular abnormalities characterized by direct communications between pulmonary arteries and veins (1). The definitive diagnosis of PAVMs relies on clinical presentation combined with imaging modalities, including contrast-enhanced computed tomography (CT), contrast echocardiography, and three-dimensional CT pulmonary angiography (3DCTPA), with conventional pulmonary angiography reserved as the gold standard. PAVMs are frequently associated with hereditary hemorrhagic telangiectasia (HHT), also known as Osler-Weber-Rendu disease (2, 3). Approximately 70% of PAVMs are associated with HHT, whereas 15%–50% of patients with HHT develop PAVMs (4).

Hemodynamically, these lesions create right-to-left shunts that may result in chronic hypoxemia and increase the risk of paradoxical embolism, stroke, and brain abscess (5). Although some patients remain asymptomatic, timely intervention is generally recommended once the diagnosis is established. With the development of minimally invasive thoracic surgery, video-assisted thoracoscopic surgery (VATS) has evolved from conventional multi-port techniques toward uniportal approaches (6–8). Consequently, VATS has become an important treatment option for pulmonary lesions and selected PAVMs (9–11).

### 1.2 Rationale and knowledge gap

Despite the increasing use of transcatheter embolization as the preferred first-line treatment for many PAVMs, surgical intervention remains a vital option for selected patients with large lesions or high risk of rupture. With the evolution of minimally invasive surgery, uni-portal video-assisted thoracoscopic surgery (UVATS) has emerged as an attractive alternative to conventional thoracotomy and multi-portal VATS. By consolidating the camera and instruments into a single 3-cm incision, UVATS minimizes intercostal nerve irritation, reduces postoperative pain, and enhances cosmetic satisfaction. However, reports describing the application of UVATS for PAVM treatment remain scarce, and clear evidence regarding temporary vascular pre-blocking techniques to optimize intraoperative safety is lacking (6–11).

Furthermore, although individual case reports have described thoracoscopic management of PAVMs, a comprehensive summary of thoracoscopic treatment strategies is lacking. In particular, evidence regarding vascular pre-blocking techniques to reduce intraoperative bleeding and embolic complications remains scarce.

### 1.3 Objectives

Herein, we report the successful management of a large asymptomatic PAVM in a 43-year-old woman using UVATS combined with pre-blocking of the right lower pulmonary artery and pulmonary vein before lesion resection. In addition, we reviewed the literature on thoracoscopic treatment of PAVMs to summarize current surgical strategies and provide further clinical insights. This manuscript was prepared according to the CARE reporting guideline.

## 2 Case presentation

Written informed consent for publication was obtained from the patient.

**FIGURE 1:**
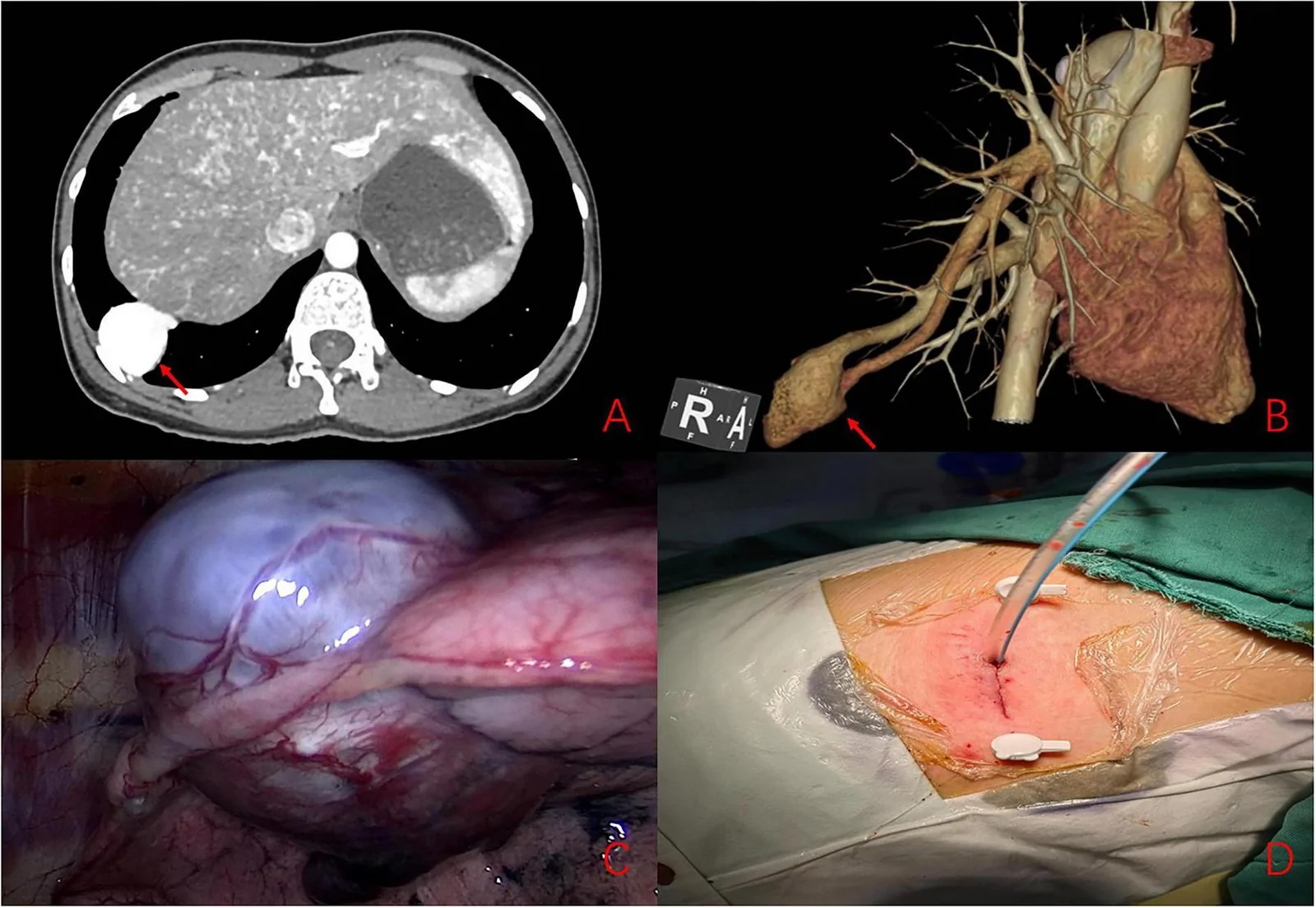
**(A)** A computed tomography scan of the chest revealed a space-occupying mass in the outer basal segment of the right lower lobe, measuring approximately 3.1 × 2.7 cm. Enhanced scan showed significant vascular enhancement, indicated by the red spiral. **(B)** 3D CT reconstruction showed marked enlargement of the mass after enhancement, with connections to both the thickened right lower pulmonary vein and the pulmonary artery of the right lower lobe, indicated by the red spiral. **(C)** Pulmonary arteriovenous malformation (PAVM) with thrombus formation on the surface of the basal segment of the right lower lung seen on video thoracoscopy. **(D)** Subcutaneous suture of the incision with titanium alloy thread.

A 43-year-old woman was admitted after a routine chest CT examination revealed a mass in the right lower lobe of the lung. The patient was asymptomatic and denied hemoptysis, dyspnea, hypertension, diabetes mellitus, cardiovascular disease, and respiratory disorders. Physical examination revealed a continuous vascular murmur at the level of the sixth rib in the right axillary region. Laboratory testing demonstrated hemoglobin of 126 g/L, partial pressure of oxygen (PO_2_) of 66 mmHg, partial pressure of carbon dioxide (PCO_2_) of 30 mmHg, pH of 7.402, and oxygen saturation of 92%. Chest CT demonstrated a well-circumscribed lesion measuring approximately 3.1 cm × 2.7 cm in the outer basal segment of the right lower lobe (RS9). Contrast-enhanced imaging showed marked vascular enhancement and communication with the pulmonary artery (Figure 1A). Three-dimensional CT reconstruction further demonstrated enlargement of the lesion after enhancement and revealed direct communication with both the enlarged right lower pulmonary vein (RLV) and the segmental pulmonary artery (RA9) (Figure 1B). Brain and upper abdominal CT examinations revealed no significant abnormalities.

**FIGURE 2:**
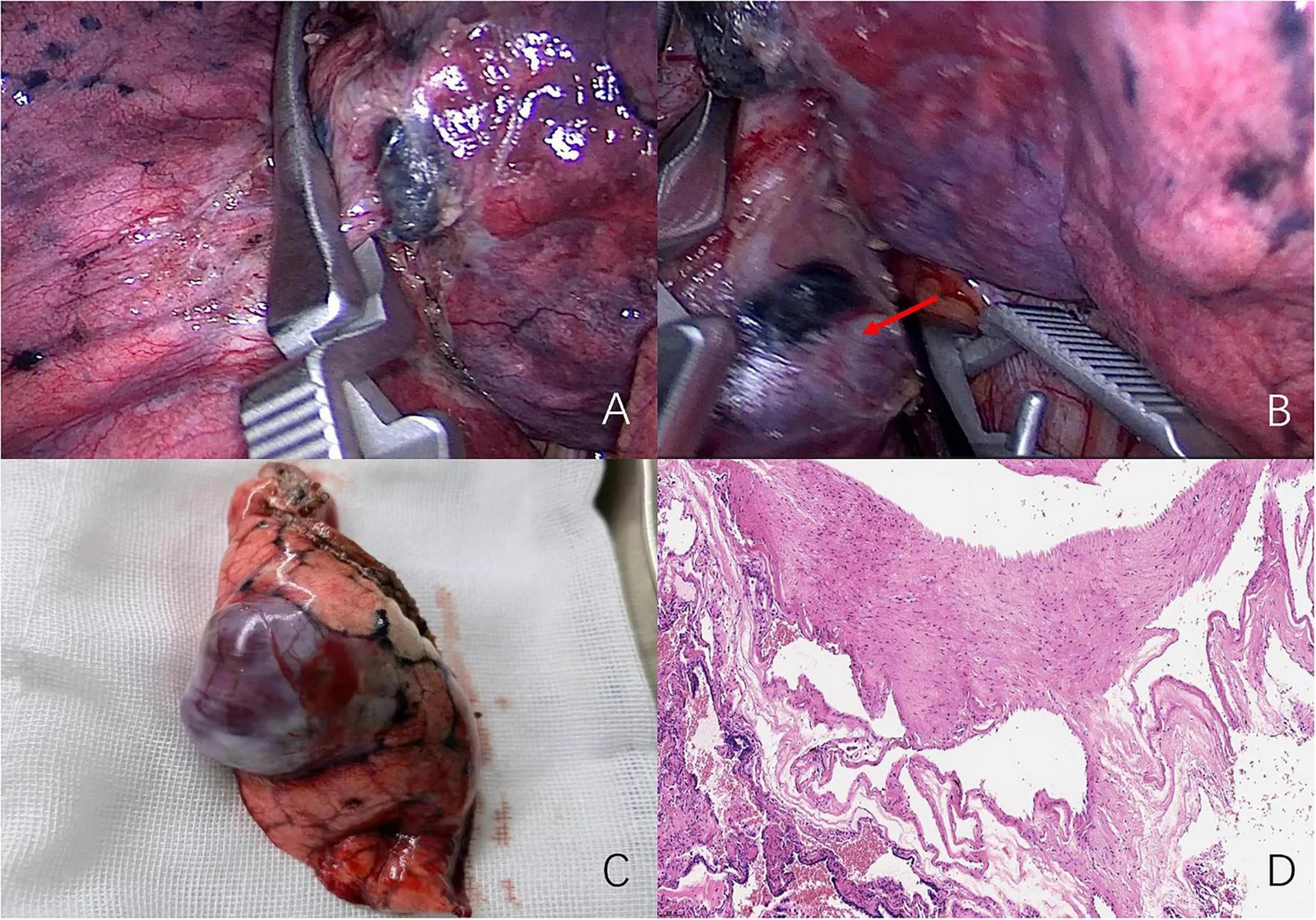
**(A)** Right lower pulmonary artery trunk pre-blocked by Concavo-Convex Tooth Hemostatic Clip (CCTHC). **(B)** After exposing the right lower pulmonary vein (RLV) by pulling with 10 silk threads, the vessels were pre-occluded using a CCTHC with the arrow pointing to the RV. **(C)** Postoperative specimen condition. **(D)** Post-operative pathology showed many irregularly enlarged blood vessels, consistent with pulmonary arteriovenous fistula changes.

The patient subsequently underwent UVATS under general anesthesia with double-lumen endotracheal intubation. A 3-cm incision was made through the fifth intercostal space between the right midaxillary and posterior axillary lines (Figure 1D). Intraoperatively, a pulsatile mass was identified in the RS9 segment (Figure 1C). After meticulous hilar dissection, the inflow and outflow portions of the right lower pulmonary artery trunk were fully exposed and temporarily occluded using a Concavo-Convex Tooth Hemostatic Clip (CCTHC) (Figure 2A). The right lower pulmonary vein was subsequently dissected, exposed, and controlled with silk traction to facilitate vascular occlusion (Figure 2B). Following vascular control, wedge resection of the lesion was performed using a linear stapling device, and the staple line was reinforced with 4-0 Prolene sutures. The specimen was removed for routine pathological examination (Figure 2C). The operation lasted 50 min without intraoperative complications. Postoperatively, oxygen saturation improved to 98% and PO_2_ increased to 82 mmHg. The chest tube was removed on postoperative day 2, and the patient was discharged on postoperative day 3. Histopathological examination demonstrated numerous irregularly dilated vascular channels, confirming the diagnosis of PAVM (Figure 2D). At the 3-month follow-up visit, the patient remained asymptomatic without recurrence or adverse events (Table 1).

**TABLE 1:** Timeline of the important event of patient.

Time	Clinical event
Admission	Chest CT identified right lower lobe mass
Preoperative evaluation	CT angiography and 3D reconstruction confirmed PAVM
Surgery	UVATS with pre-blocking of right lower pulmonary artery and vein
Postoperative day 2	Chest tube removed
Postoperative day 3	Discharged
3-month follow-up	No recurrence or complications

## 3 Discussion

### 3.1 Key findings

The present case demonstrates that UVATS combined with temporary pre-blocking of both feeding and draining pulmonary vessels is a feasible and safe strategy for the treatment of large PAVMs. Complete lesion resection was achieved through a minimally invasive approach, and the patient experienced rapid postoperative recovery without recurrence or complications during follow-up.

### 3.2 Strengths and limitations

A major strength of this report is the description of a practical, reproducible vascular control strategy using temporary pre-blocking, which minimizes the inherent risks of severe intraoperative hemorrhage and paradoxical systemic embolization during manipulation of large vascular anomalies. However, several limitations must be acknowledged. First, due to the nature of a single-case design, these findings cannot be generalized to all anatomical variants of PAVMs. Second, our follow-up period was limited to 3 months, which prevents the evaluation of long-term surgical efficacy, late recanalization, or long-term recurrence rates. Third, this study does not allow a direct head-to-head comparison between UVATS and catheter-based embolization therapy under identical conditions. Consequently, randomized clinical trials or large-scale multicenter longitudinal studies are required to establish objective comparative guidelines.

### 3.3 Comparison with similar research

Current treatment options for PAVMs include transcatheter embolization and surgical resection. The use of coils and occlusion devices is gradually replacing traditional surgery and is expected to remain the preferred treatment strategy for many patients (12, 13). However, embolization may be associated with complications including air embolism, coil migration, vascular injury, pulmonary infarction, and pulmonary hypertension (14, 15). Moreover, embolization of small lesions may be technically challenging, and the risk of neurological complications appears unrelated to lesion size (16, 17). Compared with thoracotomy, VATS offers reduced surgical trauma and faster postoperative recovery. Specifically, Uniportal VATS (UVATS) was selected over conventional open thoracotomy and multi-portal VATS due to its significant procedural advantages. Compared to thoracotomy, UVATS minimizes chest wall trauma, preserves intercostal muscle integrity, reduces postoperative pain, and accelerates functional recovery. Compared to multi-portal VATS, UVATS consolidates instruments and the camera into a single 3-cm incision, further reducing intercostal nerve irritation and improving cosmetic satisfaction. Given that the lesion was located peripherally in the outer basal segment (RS9), UVATS offered optimal visualization and accessibility. Bakhos et al. (14) reported symptom resolution following thoracoscopic surgery in a patient who had previously undergone repeated embolization procedures, whereas Heitmann et al. (18) described successful surgical treatment of a patient with recurrent brain abscess after embolization. Similarly, Litzler et al. (19) reported favorable outcomes using a combined embolization and thoracoscopic approach. Collectively, these reports suggest that thoracoscopic surgery remains an important treatment option in selected patients.

### 3.4 Explanations of findings

To better understand current surgical practice, we reviewed 21 published reports of thoracoscopic treatment for PAVMs and pulmonary arteriovenous fistulas. Among these patients, seven were male and 14 were female (Table 2). Most presented with symptoms such as epistaxis, dyspnea, headache, visual disturbances, chest pain, hemoptysis, or dizziness; some had a history of asthma (1, 2, 4, 5, 14, 19–31). Four pregnant patients were identified, three of whom required emergency surgery for hemothorax (23, 27, 30). Pregnancy-associated increases in blood volume, cardiac output, and progesterone levels may contribute to vessel dilation and rupture, highlighting the need to consider PAVMs when evaluating spontaneous hemothorax during pregnancy. Notably, four previously reported patients exhibited audible chest murmurs (2, 3, 22, 30), a finding that was also present in our patient, emphasizing the value of careful physical examination.

**TABLE 2:** Literature review of thoracoscopic surgery for pulmonary arteriovenous malformation (PAVM).

References	Sex	Age (year)	Symptom	CAM	Associated diseases or past medical history	Location	Diagnosis	NS	IT	Operation	PC	PFU (m)
Iida et al. (5)	M	54	Recurrent epistaxis and respiratory distress	NA	NA	BL	CT/3DCT	N	No	Wedge resection	No	6
Watanabe et al. (2)	F	45	Headache and left homonymous hemianopia	Yes	Brain abscess	RML	CT/CTA	S	No	Wedge resection	No	NA
Bakhos et al. (14)	F	38	Hemoptysis, syncope, epistaxis	NA	Osler-Weber-Rendu syndrome	LU	CT	N	Yes	Lobectomy	No	9
Bakhos et al. (14)	F	67	Chest pain	NA	PAVM	LL	CT	N	Yes	Wedge resection	No	NA
Litzler et al. (19)	F	35	Sudden left thoracic pain and dyspnea	NA	Hereditary and recurrent epistaxis and lip telangiectasia	L	CT/CTA	N	Yes	NA	No	24
Thung et al. (20)	F	64	Massive hemoptysis	NA	NA	RML	X-Ray/CTA	NA	No	Lobectomy	NA	6
Forster and Schulte (21)	F	41	Coughing up blood and dizziness	NA	NA	NA	NA	NA	NA	Wedge resection	NA	–
Ito et al. (22)	M	66	Dyspnea and left hemianopia	Yes	TIA	LL	X-Ray/CT	S	No	Pulmonary Vein Clamp	No	NA
Han et al. (23)	F	52	Exertional dyspnea and occasional chest pain	NA	NA	LU	CT/3DCT	S	NA	Wedge resection	No	NA
Heitmann et al. (18)	F	58	NA	NA	Brain abscess	LL	CTPA	N	Yes	Lobectomy	No	16
Wang et al. (24)	F	32	Sudden-onset dyspnea and backache	NA	Pregnancy status	RML	CTA	N	No	Wedge resection	No	NA
Kohno et al. (3)	F	15	NA	Yes	Polycythemia	RUL	CT\CTA	N	Yes	Arterial ligation	NA	NA
Li et al. (25)	NA	13	Mild hemoptysis and dyspnea	NA	NA	LL	CT/CTA/3DI	S	No	Lobectomy	No	6
Sano and Tsuchiya (1)	F	37	Dyspnea	NA	NA	BL	CT	NA	No	Removal and ligation of blood vessels	No	24
Matsutani et al. (26)	M	25	Chest pain	NA	Birt-Hogg-Dube	LL	CT	N	No	NA	NA	NA
Wang et al. (27)	M	15	Difficulty breathing and chest pain	NA	Left hemothorax	BL	CT	N	No	Wedge resection	No	NA
Shiiya et al. (4)	M	64	Dyspnea	NA	Brain abscess, left hemothorax, fibrillation	LL	CT/3DCT	S	No	Wedge resection	No	12
Kim (28)	F	24	Chest pain and dyspnea	NA	Right hemothorax, pregnancy status	RU	CT	S	No	Wedge resection	Yes	8
Li et al. (29)	M	44	Hemoptysis	NA	TIA	RL	CT/3DCT	N	Yes	Lobectomy	No	3
Duan et al. (30)	F	25	Chest pain, dizziness, and dyspnea	Yes	Left hemothorax, pregnancy status	LU	CTA	NA	No	Wedge resection	No	NA
Naito et al. (31)	M	34	Chest pain and dyspnea	NA	Right hemothorax, Pregnancy status, telangiectases, epistaxis	RM	CT	S	NA	Wedge resection	No	NA
Shien et al. (15)	F	60	No	NA	Vulvar carcinoma, MALT	LL	CT	S	No	Lobectomy	No	2

M, male; F, female; NA, information not available; CAM, chest auscultation murmur; TIA, transient ischemic attack; MALT, mucosa-associated lymphoid tissue; UL, left upper lobe, LL, left lower; RU, right upper, RL, right lower; BL, bilateral lungs; M, middle; RULL, right upper and lower; 3DCT, 3-dimensional CT; CT, computed tomography; CTA, CT angiography; NL, number of lesions; M, multiple; S, single; IT, interventional therapy; PC, postoperative complications; PFU, postoperative follow-up; m, month.

Thoracoscopic surgical techniques reported in the literature include wedge resection, segmentectomy, lobectomy, pneumonectomy, and, in rare circumstances, lung transplantation. Sano and Tsuchiya (1) described thoracoscopic ligation and resection of multiple abnormal vessels while preserving normal lung tissue; however, this approach may be unsuitable for peripheral multiple lesions and may increase concerns regarding bleeding or thrombosis. Wang et al. (27) reported successful thoracoscopic wedge resection in a patient with multiple ruptured fistulas, whereas Shien et al. (15) described symptom improvement following thoracoscopic lobectomy. Surgical decision-making should therefore be individualized according to lesion size, number, location, and proximity to the pulmonary hilum.

In the present case, three-dimensional reconstruction demonstrated a large fistulous connection exceeding 3 cm between RA9 and the right inferior pulmonary vein. Because direct manipulation of the lesion carried a substantial risk of bleeding, we first performed complete hilar dissection and temporary occlusion of both the right inferior pulmonary artery and vein before resection. Pulmonary vascular pre-occlusion is commonly used during complex thoracic procedures and has been shown to reduce intraoperative hemorrhage (32, 33). We believe this strategy not only minimizes bleeding risk but may also reduce the possibility of embolic material entering the systemic circulation during surgical manipulation.

### 3.5 Implications and actions needed

Our experience demonstrates the feasibility of UVATS in patients with large PAVMs accompanied by markedly dilated vessels, suggesting that temporary vascular pre-blocking may improve surgical safety. Future multicenter studies involving larger patient cohorts and longer follow-up periods are required to validate the efficacy and reproducibility of this surgical strategy and to clarify its role relative to embolization-based treatment.

## 4 Conclusion

This case provides additional evidence supporting the use of UVATS in selected patients with PAVMs. Temporary pre-blocking of the pulmonary artery and pulmonary vein before lesion resection may enhance operative safety and facilitate successful minimally invasive treatment of large vascular malformations. Further studies are warranted to confirm these findings and determine their long-term clinical applicability.

## Data Availability

The original contributions presented in this study are included in this article/supplementary material, further inquiries can be directed to the corresponding authors.
